# Neural Basis of Psychological Growth following Adverse Experiences: A Resting-State Functional MRI Study

**DOI:** 10.1371/journal.pone.0136427

**Published:** 2015-08-20

**Authors:** Takashi X. Fujisawa, Minyoung Jung, Masahiko Kojima, Daisuke N. Saito, Hirotaka Kosaka, Akemi Tomoda

**Affiliations:** 1 Research Center for Child Mental Development, University of Fukui, Fukui, Japan; 2 Division of Developmental Higher Brain Functions, United Graduate School of Child Development, University of Fukui, Fukui, Japan; 3 Biomedical Imaging Research Center, University of Fukui, Fukui, Japan; 4 Department of Neuropsychiatry, Faculty of Medical Sciences, University of Fukui, Fukui, Japan; Chiba University Center for Forensic Mental Health, JAPAN

## Abstract

Over the past decade, research on the aftereffects of stressful or traumatic events has emphasized the negative outcomes from these experiences. However, the positive outcomes deriving from adversity are increasingly being examined, and such positive changes are described as posttraumatic growth (PTG). To investigate the relationship between basal whole-brain functional connectivity and PTG, we employed resting-state functional magnetic resonance imaging and analyzed the neural networks using independent component analysis in a sample of 33 healthy controls. Correlations were calculated between the network connectivity strength and the Posttraumatic Growth Inventory (PTGI) score. There were positive associations between the PTGI scores and brain activation in the rostral prefrontal cortex and superior parietal lobule (SPL) within the left central executive network (CEN) (respectively, *r* = 0.41, *p* < 0.001; *r* = 0.49, *p* < 0.001). Individuals with higher psychological growth following adverse experiences had stronger activation in prospective or working memory areas within the executive function network than did individuals with lower psychological growth (*r* = 0.40, *p* < 0.001). Moreover, we found that individuals with higher PTG demonstrated stronger connectivity between the SPL and supramarginal gyrus (SMG). The SMG is one of the brain regions associated with the ability to reason about the mental states of others, otherwise known as mentalizing. These findings suggest that individuals with higher psychological growth may have stronger functional connectivity between memory functions within the CEN and social functioning in the SMG, and that their better sociality may result from using more memory for mentalizing during their daily social interactions.

## Introduction

Research on the aftereffects of stressful or traumatic events has traditionally emphasized the negative outcomes from these experiences, as well as the therapeutic interventions for posttraumatic psychopathology [1–3]. Evidence gathered during the past decade has increasingly suggested that positive outcomes can derive from adversity and other negative events. These studies have included a systematic examination of the psychological domains for the positive outcomes and their associations with other social or psychological factors [4–5]. Such positive changes have been described as posttraumatic growth (PTG), a term coined by Tedeschi and Calhoun [6]. Although the psychological mechanism underlying the growth derived from distress or adversity has been discussed, currently no consensus has been reached regarding the relationship between adversity and positive outcomes [7]. Furthermore, studies on PTG tend to focus on the psychological phenomena rather than on the neurological mechanisms, thus the neural mechanisms underlying PTG remain unclear.

Previous neurological studies on traumatic events and posttraumatic stress disorder (PTSD) focused on the neural basis of the negative outcomes (e.g., [8]), rather than on the PTG following such an event. The only study to directly examine the neural basis of PTG used electroencephalography and found an evident association between left frontal brain activity and PTG in survivors of severe motor vehicle accidents [9]. Additionally, a recent structural magnetic resonance imaging (MRI) study suggested that a greater perception of growth as a primary element of their psychological well-being is associated with increased insular volume [10]. However, to the best of our knowledge, no study has used functional MRI (fMRI) techniques to characterize the functional correlates of PTG. We expected that accurate quantitative network prediction of PTG would be informed by functional alterations within a highly distributed network of regions that includes the prefrontal cortices, amygdala, and hippocampus. However, it may be difficult to measure a person’s psychological traits like PTG using fMRI by performing some specific behavioral tasks.

Resting-state fMRI (rs-fMRI) is a superior method for characterizing the baseline brain activity [11] that occurs during periods where participants are not performing a task, which can minimize the effects of external stimuli [12]. A previous study showed that task-dependent changes in brain activity comprise less than 5% of the total activity, while most of the brain’s resources are related to task-independent, spontaneous neural activity; hence, rs-fMRI is the best method for evaluating this type of activity [13]. Although spontaneous neural activity has a functionally homogeneous aggregate of anatomically independent brain areas, also called resting-state networks (RSNs), such a unique relationship is just an approximation and several functional subunits may exist. This is particularly true for the default mode network (DMN), where functional subunits can be discriminated in terms of their specific activity patterns [14], their relevance within the local or global connectivity network [15], or the influence they exert on other RSNs [16]. Independent component analysis (ICA) is a statistical approach that investigates whole-brain functional connectivity by separating signals on a subject-specific level into spatiotemporal components.

This study hoped to expand the limited knowledge on the neural basis of PTG, especially the RSNs, by using rs-fMRI to address the mechanisms underlying the emergence of PTG following adverse experiences. In order to take into account the presence of functional subunits within each RSN, a voxel-based approach was used. To study the relationship between basal brain activity and PTG, we employed ICA to explore which RSN might be differentially activated in normal adults who exhibit higher levels of PTG compared to those with lower levels of PTG. We predicted that PTG is related to increased functional connectivity between regions of the brain that are associated with cognition and other social affective functions.

## Methods

### Participants

A total of 33 right-handed, healthy volunteers (21 females, 12 males; mean age = 21.9 ± 5.7 years; age range, 18–48 years) were recruited from the local community and completed the study. Participants with Axis I disorders and Axis II disorders, a history of major medical or neurological illnesses, including epilepsy or significant head trauma, or a lifetime history of alcohol or drug dependence, as determined using the structured clinical interview for the Diagnostic and Statistical Manual of Mental Disorders (DSM-IV), were excluded [17–19]. The study protocol was approved by the ethics committee of the University of Fukui, and was conducted in accordance with the Declaration of Helsinki. After having the study completely explained to them, all participants provided written informed consent.

### Psychological measures

#### Types of stressful life events

To identify what types of stressful or traumatic life events participants had experienced as adversity, we prepared a list of 10 options corresponding to representative stressful events as follows: death of family/close friend, parental divorce, domestic discord, academic failure, domestic economic issues, bullying, interpersonal conflicts, serious illness or injury, accident or disaster, and other. Then, we asked the participants to select the most suitable item from among the list of options.

#### PTG

To quantify participants’ PTG, we used the Japanese version of the Posttraumatic Growth Inventory (PTGI) [20], an instrument used to assess the positive outcomes of people who have experienced traumatic or stressful events. The original PTGI is a 21-item scale that measures the degree of positive change experienced in the aftermath of an identified traumatic event [5]. The Japanese version has been confirmed to have acceptable validity and reliability of internal consistency (Cronbach’s alpha = 0.90) with Japanese samples [20]. The PTGI consists of four subscales: relating to others, new possibilities, personal strength, and spiritual change and appreciation of life. Each item was rated on a six-point Likert scale, ranging from 0 (not at all) to 5 (a very great degree). Higher scores imply higher levels of PTG.

#### Posttraumatic distress

To assess the posttraumatic symptoms experienced by each participant, we used the Japanese version of the Impact of Event Scale-Revised (IES-R) [21]. The original IES-R is a widely used 22-item scale that measures traumatic symptoms and is comprised of three subscales: intrusion, avoidance, and hyperarousal [22–23]. The Japanese version of the IES-R is in accordance with the original English version in terms of its items and subscales. Each item was rated on a five-point Likert scale, ranging from 0 (not at all) to 4 (a very great degree). Higher scores imply higher levels of traumatic symptoms. The Japanese version of the IES-R has demonstrated satisfactory validity and test-retest reliability (*r* = 0.86) [21].

Similarly, depression symptom severity was assessed with the Japanese version of the Beck Depression Inventory (BDI) because previous studies have suggested a positive association not with PTSD symptoms, but with depression symptoms, implying that depression symptoms play a critical role in PTG [24]. The original version is a validated measure of depression and has an established reliability [25]; participants rated each item on a scale of 0 (strongly disagree) to 3 (strongly agree).

### Image acquisition

Imaging was performed using a 3-Tesla scanner (Discovery MR 750; General Electric Medical Systems, Milwaukee, WI, USA) and a 32-channnel head coil. Resting-state data were acquired with a T2*-weighted gradient-echo echo-planar imaging (EPI) sequence and each volume consisted of 40 slices, with a thickness of 3.5 mm and a 0.5-mm gap, in order to cover the entire brain. The time interval between two successive acquisitions of the same slice (repetition time; TR) was 2300 ms, with an echo time (TE) of 30 ms and a flip angle of 81°. The field of view was 192 × 192 mm, and the matrix size was 64 × 64, yielding volume dimensions of 3 × 3 mm. A total of 201 volumes were acquired for an imaging time of 7 min 42 s. The participants were instructed to stay awake, but to close their eyes and think of nothing in particular. A T1-weighted anatomical dataset was obtained from each subject by using a magnetization-prepared rapid acquisition gradient echo sequence (voxel size 1 × 1 × 1 mm, TE = 1.996 ms, TR = 6.38 ms, TI = 600 ms, flip angle = 11°, total scan time = 4 min 50 s).

### fMRI data preprocessing

fMRI data were analyzed using SPM8 (http://www.fil.ion.ucl.ac.uk/spm/), a data processing assistant, with the following steps. First, the initial 10 volumes were discarded, and then slice-timing correction was performed, followed by spatially realigning the remaining 191 volumes to the mean volume. The signal from each slice was realigned temporally to that obtained from the middle slice using sinc interpolation. The EPI and structural scans were co-registered and normalized to the T1 standard template in Montreal Neurological Institute (MNI) space using linear and nonlinear transformations. The normalized images were spatially smoothed with a 6-mm Gaussian kernel. Next, the linear trend in the time series was removed and temporal bandpass filtering (0.0078–0.08 Hz) was performed to reduce the effects of low-frequency drift and high-frequency noise [26]. The non-neural noise in the time series was controlled, and several sources of spurious variance, six parameters, white matter signals, and cerebrospinal fluid signals were removed from the data through linear regression [27]. We computed the mean frame-to-frame root mean square (RMS) motion and frame-wise displacement (FD) obtained in the realignment process for each participant to investigate the relationship between head motion and the PTGI scores.

### ICA

Group spatial ICA was conducted for all 30 participants using the Group ICA/IVA of fMRI Toolbox (GIFT v2.0a, mialab.mrn.org) program with the infomax algorithm [28]. Data were decomposed into 30 components for each participant. Principal component analysis was executed for data reduction in all participants, and the decomposition was alternated with data across all participants. The individual components were calculated with back reconstruction performed with the offset and was computed using the mean component maps, which were subtracted from the individual component maps by computing the offset using the mean component maps. The mean ICA components were transformed to *z*-values with the time course of the relevant component. Eight components corresponding to the functional networks described in previous studies were selected.

### Relationship between RSNs and PTG

To investigate the relationship between RSNs and PTG scores, we performed multiple regression analysis (MRA) using SPM, and resting-state fMRI software (DPARSF) [29]. Sex and age were included as covariates in the model due to their potential confounding effects. In addition, the BDI score was included as a covariate because the score was negatively correlated with the PTGI score, thus the effect of the score on PTG needed to be excluded. The statistical threshold for contrasts was voxel-level *p* < 0.001 uncorrected for height and cluster-level *p* < 0.05 corrected for multiple comparisons. Results of the MRA were masked by the results of the one-sample *t*-tests from the ICA (threshold for contrasts was *p* < 0.005). In addition, we performed seed-based analysis to clarify the RSNs from the results of the multiple regressions with the PTGI scores from the ICA. The seeds for the seed-based analyses were localized in the results of the multiple regressions with the PTGI scores from the ICA using the Marsbar software package (http://marsbar.sourceforge.net). The mean time course of all voxels in each seed measured was used to calculate the voxel-wise linear correlations (Pearson’s correlations) for the whole brain, and the individual *r* values were then normalized to *z* values using Fisher’s *z* transformation.

## Results

### Demographic characteristics

Three participants (2 males and 1 female) had to be removed from the analysis due to exclusion criteria related to excessive maximum head motion (over 2.0 mm, 2.0°, and 0.20 mean FD) during scanning. The characteristics of the 30 participants that were included in the analyses are listed in Table 1. There were no significant relationships between the head movement measures (mean frame-to-frame RMS motion, mean FD) and the PTGI (*p* = 0.25; *p* = 0.45), BDI (*p* = 0.23; *p* = 0.67), or IES-R (*p* = 0.61; *p* = 0.86).

**Table 1:** Demographic data.

Measure	Participants (n = 30)	
Sex (Female/Male)	20/10	–
Handedness: Right/Left^a^	30/0	–
	**Mean**	**SD**
Age	21.5	3.4
RMS Mean Displacement (mm)	0.036	0.017
Mean FD (mm)	0.123	0.035

^a^Assessed by the Edinburgh handedness inventory.

**Abbreviations:** SD, Standard Deviation; RMS, Root Mean Square; FD, Frame-wise Displacement.

### Psychological characteristics

The frequency and proportion of each type of stressful life event experienced as adversity are shown in Table 2. Among the 30 participants, the most stressful or traumatic events they had experienced were interpersonal conflicts (33.3%), academic failure (20.0%), death of family/close friend (16.7%), bullying (16.7%), serious illness or injury (10.0%), and domestic discord (3.3%). None of the participants selected parental divorce, domestic economic issues, or accident or disaster as their adverse experiences in the present study.

**Table 2:** Type and frequency of stressful life events experienced as adversity.

Category	Event type	n (%)
1	Death of family/close friend	5 (16.7)
2	Parental divorce	0 (0.0)
3	Domestic discord	1 (3.3)
4	Academic failure	6 (20.0)
5	Domestic economic issues	0 (0.0)
6	Bullying	5 (16.7)
7	Interpersonal conflicts	10 (33.3)
8	Serious illness or injury	3 (10.0)
9	Accident or disaster	0 (0.0)
10	Other	0 (0.0)

The mean values and standard errors of each psychological measure are presented in Table 3, together with the correlations between the measures. The analyses yielded significant relationships between the BDI scores and the PTGI or IES-R scores, respectively, but we did not find a significant relationship between the PTGI and IES-R scores, which has often been reported by previous studies [4,30].

**Table 3:** Means, standard deviations, and intercorrelations between the psychological measures.

			Correlation	
Measure	Mean	SD	IES-R	BDI
PTGI	69.7	22.2	-0.19^n.s.^	-0.40*
IES-R	15.2	13.8		0.67**
BDI	5.4	5.0		

*p < 0.05

** *p* < 0.01, n.s., not significant.

**Abbreviations:** SD, Standard Deviation; PTGI, Posttraumatic Growth Inventory; IES-R, Impact of Event Scale–Revised; BDI, Beck Depression Inventory.

### ICA

**Fig 1:**
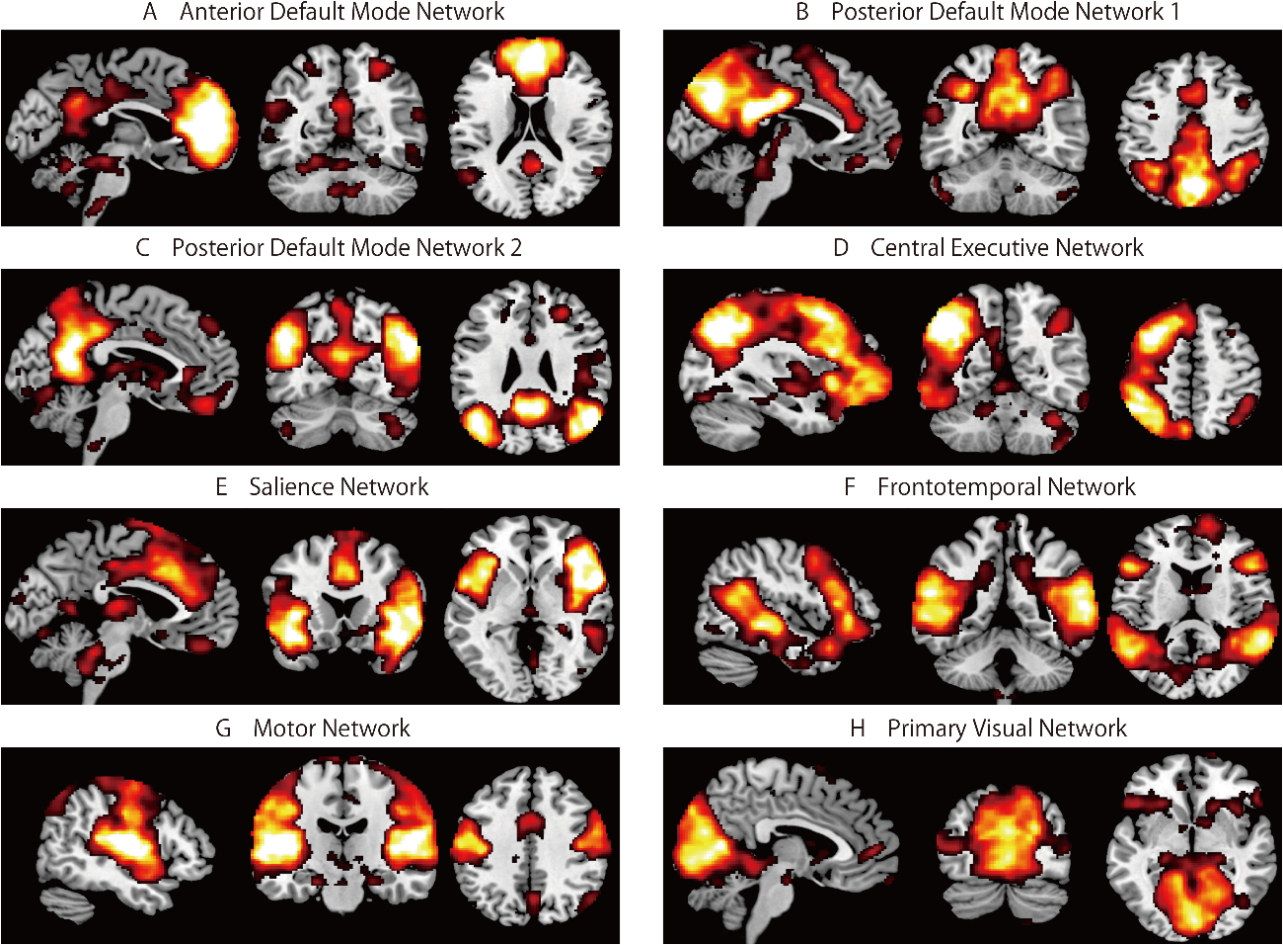
Results of the independent component analysis. Average components for all participants. Maps show the following independent components: A, anterior default mode network (DMN); B, posterior DMN1; C, posterior DMN2; D, central executive network; E, salience network; F, frontotemporal network; G, motor network; and H, primary visual network. The statistical threshold for the maps was voxel-level *p* < 0.001 uncorrected for height and cluster-level *p* < 0.05 corrected for multiple comparisons. The coordinates for the panels are as follows: A (4, -52, 32), B (4, -58, 42), C, (4, -65, 27), D (-34, -54, 52), E (-4, 18, -2), F (46, -44, 14), G (48, -16, 39), and H (4, -78, -4).

Eight components corresponding to functional networks from previous studies [31–32] were selected (Fig 1).

We found three components resembling the DMN, with key brain regions in the medial prefrontal cortex (MPFC) and posterior cingulate cortex (PCC), which are involved in social cognition and self-relevant processing [33–35]. The spatial template matching procedure was performed using the DMN template provided in the GIFT program. Three components were selected based on the ranked highest correlation values (anterior DMN: 0.48, posterior DMN1: 0.61, posterior DMN2: 0.40). The other five components of the RSN were identified via ICA, as follows. The central executive network (CEN), with key brain regions including the dorsolateral PFC and PCC, is involved in information manipulation and decision-making behaviors [36]. The salience network, with key brain regions in the dorsal anterior cingulate cortex (ACC) and insular cortex, is involved in mediating the functions of other networks [37–39]. The frontotemporal network, with key brain regions in the superior temporal gyrus and inferior frontal gyrus, is involved in memory encoding and performance [40]. The motor network, with key brain regions in the precentral gyrus and supplementary motor area, is involved in motor function [41]. Finally, we also found the primary visual network, which has key brain regions in the occipital lobe and is involved in visual functions [42].

### Correlation with PTG

**Fig 2:**
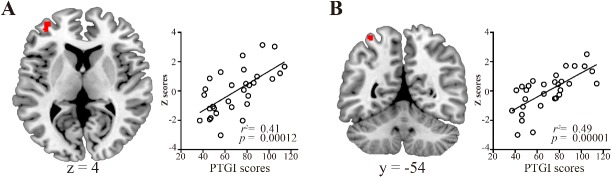
Results of the multiple regression analysis between the central executive network and the scores on the posttraumatic growth inventory (PTGI). Brain regions showing positive correlations between the PTGI scores and the strength of the central executive network activity as determined by multiple regression analysis. The scatter plots show the association between the PTGI scores and the strength of the central executive network activity. A, rostral prefrontal cortex; B, superior parietal lobule. The statistical threshold for the contrasts was voxel-level *p* < 0.001 uncorrected for height and cluster-level *p* < 0.05 corrected for multiple comparisons.

Results of the MRA showed that the PTGI scores were significantly positively correlated with the strength of the brain activity in the rostral prefrontal cortex (rPFC) and superior parietal lobule (SPL) in the CEN (Fig 2; Table 4). There were no significant correlations with the other networks.

**Table 4:** Brain regions showing correlations between the central executive network and the scores on the posttraumatic growth inventory.

		MNI coordinates				Cluster size
Region (Brodmann area)	Side	x	y	z	Z-score	kE (voxels)
Positive correlation						
Rostral prefrontal cortex (10)	L	-34	54	4	3.81	62
Superior parietal lobule (7)	L	-34	-54	66	4.38	36
Negative correlation						
None						

The statistical threshold for contrasts was voxel-level *p* < 0.001 uncorrected for height and cluster-level *p* < 0.05 corrected for multiple comparisons.

**Abbreviations:** MNI, Montreal Neurological Institute.

**Fig 3:**
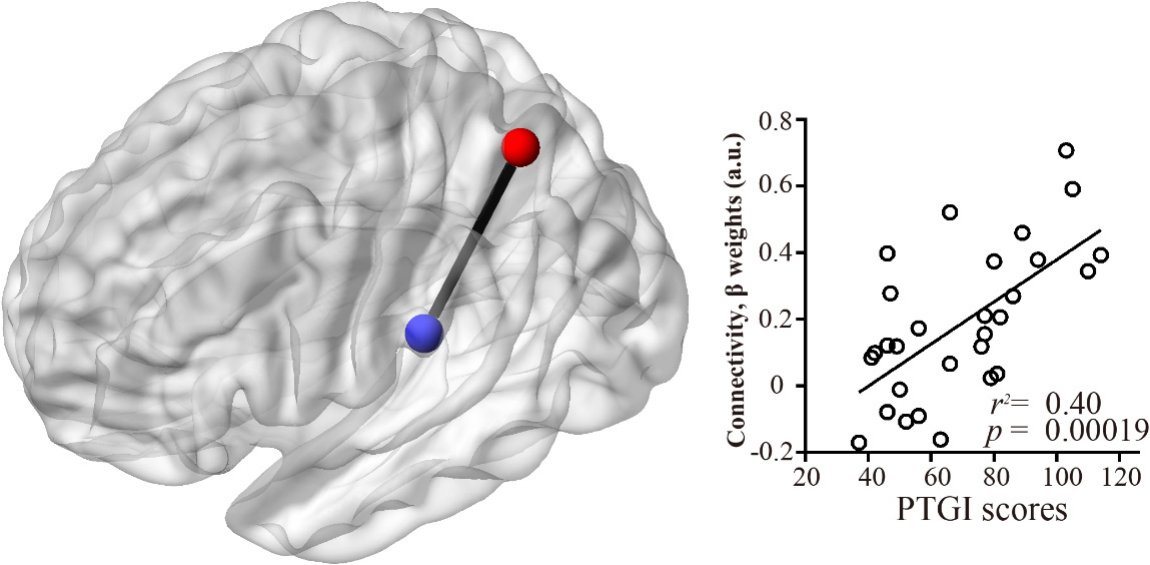
Brain regions showing positive correlations between the scores on the PTGI and the strength of the functional connectivity from the superior parietal lobule seed. Brain regions showing positive correlations between the PTGI scores and the strength of the activity in the RSNs from the superior parietal lobule seed as determined by multiple regression analysis. The statistical threshold for the contrasts was voxel-level *p* < 0.001 uncorrected for height and cluster-level *p* < 0.05 corrected for multiple comparisons. The scatter plots show the associations between the PTGI scores and the strength of the functional connectivity between the superior parietal lobule and superior temporal gyrus.

In the seed-based analysis, PTGI scores were significantly positively correlated with the strength of the functional connectivity between the SPL seed and supramarginal gyrus (SMG) (Fig 3; Table 5). No significant correlations were found between the PTGI scores and the strength of the RSNs in the rPFC seed.

**Table 5:** Brain regions showing correlations between the strength of the resting-state networks in the seed-based analysis and the scores on the posttraumatic growth inventory.

		MNI coordinates				Cluster size
Region (Brodmann area)	Side	x	y	z	Z-score	kE (voxels)
Seed: middle frontal gyrus						
Positive correlation						
None						
Negative correlation						
None						
Seed: superior parietal lobule						
Positive correlation						
Supramarginal gyrus (40)	L	-54	-26	24	3.62	243
Negative correlation						
None						

The statistical threshold for contrasts was voxel-level *p* < 0.001 uncorrected for height and cluster-level *p* < 0.05 corrected for multiple comparisons.

**Abbreviations:** MNI, Montreal Neurological Institute.

## Discussion

The aim of the present study was to examine the relationship between the brain activity in RSNs and the individual differences in psychological growth following adverse experiences in healthy adult subjects. The results revealed a positive association between the PTGI score and activity in the rPFC and SPL within the left CEN. This result suggests that individuals with higher psychological growth following adversity have stronger activity in the rPFC and SPL within the left CEN than do individuals with lower psychological growth. In addition, by using the seed-based analysis, we found a positive association between the PTGI score and the functional connectivity between the SPL and SMG. Interestingly, the SMG is suggested to be one of the brain regions involved in reasoning about the mental states of others, also known as mentalizing [43]. These findings suggest that individuals with higher psychological growth have higher functional associations between the SPL, as a part of the executive network, and the SMG, which may play a significant role in social interaction.

Although the neural correlates of the negative consequences after trauma such as PTSD have been investigated, the neural correlates of individual differences in PTG following adverse experiences were unclear. Some previous studies have suggested that PTG is associated with executive functions or cognitive control [44], whereas people with PTSD have significantly impaired executive functions [45] and alterations in functional connectivity within the CEN [46]. These findings are in line with our results showing increased brain activation in the CEN (including the rPFC and SPL) in individuals with higher PTGI scores. Moreover, it is important to note that these associations between PTG and regions within the CEN were found after excluding any covariate effects induced by depressive symptoms, and thus the associations likely reflect the positive outcomes caused by psychological growth. To the best of our knowledge, the present study is the first to demonstrate an association between psychological growth following adverse experiences and particular functional regions within the RSNs in healthy human adults.

We found a positive association between the PTGI score and resting-state brain activity within the CEN in the left rPFC. One possible explanation for the activation in the left rPFC may be related to the cerebral asymmetry and valence hypothesis [47–48]. One previous study examined the relationship between frontal brain asymmetry and the subjective perception of PTG after severe motor vehicle accidents using resting-state electroencephalography measurements and found significantly increased left frontal activation related to PTG [9]. The authors assumed that positive affective styles associated with higher left frontal brain activity may be involved in the process and outcome of PTG [9]. Similarly, it is possible that the activation we observed in the left rPFC within the CEN, which mediates positive affect, facilitated the achievement of PTG.

The effects of memory may be another potential explanation for the activation we observed in the lateral rPFC. Although past research on the function of the lateral aspects of the rPFC has suggested that it is associated with episodic memory retrieval [49–50], recent studies indicate that the left lateral rPFC is critically important for the focus of attention in prospective memory [51]. Prospective memory denotes the capacity to remember to carry out an intention after a delay (e.g., posting a letter) while being immersed in a distracting ongoing activity (e.g., commuting to work) [52–53]. One of the significant psychological changes that occurs with PTG includes a change in priorities or a new path in life, where prospective memory may play an important role in a person’s perspective of time or the sensation of a finite lifespan. Although there is no direct evidence that PTG has positive effects on a person’s memory, as we have already discussed, it may be that the activation we observed in the rPFC within the CEN was a reflection of some functional alterations in prospective memory caused by PTG.

For a few decades, it has been suggested that the SPL plays significant roles in various executive functions including continuous updating, order memory, and information manipulation [54], and that it contributes to episodic memory [55]. A more recent study also suggested that the SPL is critically important for manipulating information in working memory [56]. Although there is no direct evidence that PTG has beneficial effects on a person’s working memory, it has been shown that patients with PTSD demonstrate parietal event-related potentials at 400–800 ms that are smaller in amplitude, which are related to working memory updating [57]. Therefore, one possible explanation for the activation we observed in the SPL may be that it was induced by the episodic memory of the traumatic or stressful events. Meanwhile, it has been suggested that ruminative thoughts in the aftermath of traumatic/stressful events play an important role in PTG [58]. In particular, it is worth noting that one study found that intrusive rumination soon after an event is positively related to PTG, but that “recent” deliberate rumination most strongly predicted participants’ current levels of PTG [59]. Thus, as discussed above, there is no direct evidence that current rumination with PTG affects a person’s working memory; however, ruminative thought may potentially explain the brain activation we observed in the SPL within the CEN.

The temporoparietal junction (TPJ) is the area of the brain where the temporal and parietal lobes meet, and is comprised of a portion of the SMG and angular gyrus. Although it is believed that the primary function of the TPJ is to integrate information from both the external environment and internal body [60], other studies have suggested that this area plays a significant role in mentalizing [41]. In particular, the left TPJ appears to be involved in reasoning about the beliefs, intentions, and desires of others [61], which are abilities that are necessary for empathizing with others. One of the significant benefits associated with PTG is “relating to others,” meaning the expansion and deepening of interpersonal relationships [5,20]. This mentalizing ability or empathy plays an important role in constructing and maintaining better relationships with others. Therefore, it is possible that the development of interpersonal relationships after PTG may result in a positive association between memory and social functioning, and their better sociality may be provided by collaborative efforts between memory function and mentalizing in daily social interactions.

There are some notable limitations of the present study. First, the size of each group in this study was too small to demonstrate a relationship between the various aspects of PTG and the strength of the RSNs. As a second limitation, the participants of the present study were healthy adults and had not experienced any extraordinary circumstances like those that would lead to PTSD (e.g., traffic accidents or natural disasters), thus their experiences and the strength of their adversity was on the same level as that of most people (e.g., the loss of a familiar person or interpersonal conflicts). Therefore, the PTG in this study may be somewhat different from common “PTG,” which is caused by exposure to extraordinary experiences, such as serious diseases [62]. Future studies are needed to clarify the differences in the RSNs between individuals with PTSD and those with PTG following a particular adverse experience. This additional research will help provide a more complete picture that may clarify the etiology of PTG.

In conclusion, these results demonstrate that the PTG score in healthy adults is positively associated with activity in the rPFC and SPL within the left CEN as part of the RSNs. Our findings suggest that individuals with higher psychological growth following adverse experiences may have stronger activation in regions related to prospective or working memory within the executive function network than do individuals with lower psychological growth. Moreover, we also found that individuals with higher PTG have stronger connectivity between the SPL and SMG, with the SMG being involved in metalizing processes. These findings suggest that individuals with higher psychological growth have a stronger connection between memory and social functioning, and that their better sociality may be provided by using more memory when mentalizing in their daily social interactions. Mentalizing is also suggested to be important for understanding one’s own emotions and behaviors, which is essential for the process of trauma recovery [63]. A better understanding of the neural basis of PTG may help establish methods for enhancing an individual’s recovery resiliency or prevent them from developing PTSD. Further studies are necessary to confirm these findings and to clarify the causality and consequences of such neurofunctional mechanisms.

## Supporting Information

S1 Dataset The demographic, psychological assessment and functional MRI data. (PDF)
