# Supplementary material for: Neural Basis of Psychological Growth following Adverse Experiences: A Resting-State Functional MRI Study
Source: PLoS One. 2015 Aug 20;10(8):e0136427. doi: 10.1371/journal.pone.0136427 (PMC4546237; doi:10.1371/journal.pone.0136427)
Supplement: S1 Dataset — (PDF) [file pone.0136427.s001.pdf]

ID	Sex	Hand	Age	Event type	PTGI	IES-R	BDI	Head Movement	Left rPFC	Left SPL	Left SMG
1	0	1	21	1	66	37	7	0	−1.0959	−0.1891	0.5222
2	0	1	21	4	110	1	3	0	1.2154	1.35	0.3454
3	0	1	26	7	46	1	5	0	0.8657	−2.0229	0.1217
4	1	1	24	8	114	0	0	0	1.6667	0.511	0.3937
5	0	1	27	8	46	21	9	0	−1.1959	0.3004	0.3985
6	1	1	48	5	72	5	6	1			
7	0	1	33	6	49	0	0	0	−1.05	−0.5428	0.1196
8	1	1	23	7	77	41	11	0	1.4609	−0.4813	0.1564
9	1	1	21	7	86	4	1	0	0.5641	1.733	0.2702
10	0	1	20	7	76	8	0	0	−2.0146	0.6143	0.1185
11	0	1	28	4	47	26	7	0	−1.8352	−1.0675	0.2784
12	0	1	19	7	56	25	6	0	1.2981	0.6877	−0.0895
13	1	1	18	6	56	22	12	0	−3.0165	−2.8169	0.1736
14	1	1	21	4	50	6	4	0	−1.18	0.0673	−0.0108
15	0	1	20	7	80	18	5	0	−0.8808	−0.248	0.3747
16	0	1	21	6	46	17	19	0	−1.9294	−1.6074	−0.0782
17	0	1	18	1	103	7	0	0	0.832	1.6945	0.7078
18	0	1	24	6	81	5	6	1			
19	1	1	21	7	94	32	5	0	3.1413	1.7146	0.38
20	0	1	19	4	89	26	12	0	0.9899	0.4977	0.4604
21	0	1	18	7	76	10	6	0	2.4321	−0.5076	0.1151
22	0	1	18	6	77	13	6	0	0.0769	1.0788	0.2115
23	1	1	19	4	92	4	0	1			
24	1	1	20	1	79	26	5	0	0.5005	0.4624	0.0244
25	0	1	20	4	37	8	4	0	−2.0095	−1.3347	−0.1693
26	0	1	18	3	105	8	1	0	3.0371	2.5195	0.5924
27	0	1	21	7	42	0	0	0	0.1275	−2.9889	0.0993
28	0	1	20	8	82	4	0	0	0.3977	0.6479	0.2074
29	1	1	20	4	63	2	0	0	−0.6981	−1.0031	−0.1605
30	0	1	25	1	81	5	3	0	−0.321	0.0612	0.0371
31	0	1	22	1	66	5	6	0	0.4013	0.4567	0.0672
32	1	1	21	6	41	37	17	0	−0.2644	0.0596	0.0854
33	1	1	21	7	52	47	9	0	−1.5158	0.3537	−0.1061
